# 1500. Chest Computed Tomography in Respiratory Asymptomatic HIV patients

**DOI:** 10.1093/ofid/ofad500.1335

**Published:** 2023-11-27

**Authors:** Gerardo A Muñeton, Juan Pablo Perdomo-Fonseca, Laura Lucia Guevara-Plata, Mariana Espinosa Murcia, Angie Tatiana Saenz, Santiago Lopez, Melissa Rueda, Hernan Vergara

**Affiliations:** Hospital MIlitar Central - Hospital de Kennedy, BOOGOTA D.C, Distrito Capital de Bogota, Colombia; Universidad Militar Nueva Granada, Bogota, Distrito Capital de Bogota, Colombia; Universidad Militar Nueva Granada, Bogota, Distrito Capital de Bogota, Colombia; Universidad Militar Nueva Granada, Bogota, Distrito Capital de Bogota, Colombia; Universidad Militar Nueva Granada, Bogota, Distrito Capital de Bogota, Colombia; Universidad Militar Nueva Granada, Bogota, Distrito Capital de Bogota, Colombia; Universidad Militar Nueva Granada, Bogota, Distrito Capital de Bogota, Colombia; Universidad Militar Nueva Granada, Bogota, Distrito Capital de Bogota, Colombia

## Abstract

**Background:**

New HIV infections in Colombia are diagnosed in AIDS stage in 39% of cases. Some pulmonary infections are not diagnosed on time because of the absence of clinical findings, worsening the prognosis of patients. In this study we evaluate the usefulness of chest computed tomography in asymptomatic patients with HIV diagnosed with a CD4 count off less than 200cell/uL.

**Methods:**

This was an analytical cross-sectional study in 2 third-level hospitals. We included patients diagnosed with HIV without respiratory symptoms and a CD4 count of less than 200 cell/uL, who where study with a chest computed tomography. We determined the proportion of anormal chest tomography, the percentage of patients going on additional tests and treatments and the definitive diagnostics found. The association between clinical and epidemiological findings with pathological alterations found in the chest CT scan was also explored.

**Results:**

34 individuals were included. Abnormal CT scans findings were found in 18 of 34 (53%) patients. Two (5.8%) were classified as micronodular, 8 (23.5%) as nodular, 3 (5.8%) as consolidation, 2 (5.8%) as masses, 3 (8.8%) as ground glass opacities and 1 (3%) as pleural effusion. All of the patients with abnormal chest CT scan required one or more additional diagnostic tests or procedures. 8 (24%) patients received new treatments after CT scan findings and results of the additional diagnostic tests. In 6 (18%) of the patients a definitive diagnosis was possible, 2 cases of tuberculosis, 1 case of non hodgkin lymphoma, 1 case of Kaposi sarcoma and 2 cases of disseminated histoplasmosis. An association between clinical manifestations and specific chest tomography results was not found.

**Conclusion:**

All HIV respiratory asymptomatic patient with CD4 count less than 200 cells/uL should be considered for chest computerized tomography scan, taking into account that at least 50% will have abnormal findings, 30% will require new treatments and 20% will have a definitive diagnosis that could not be possible to find without the chest tomography.

**Disclosures:**

**GERARDO A. MUÑETON, Infectious diseases and Internal Medicine Specialist**, PFIZER: Advisor/Consultant

